# Supplementary material for: Identifying and Evaluating Young Children with Developmental Central Hypotonia: An Overview of Systematic Reviews and Tools
Source: Healthcare (Basel). 2024 Feb 18;12(4):493. doi: 10.3390/healthcare12040493 (PMC10887882; doi:10.3390/healthcare12040493)
Supplement: Supplementary file 1 [file healthcare-12-00493-s001.zip › healthcare-2846637-supplementary.pdf]

Supplementary Materials S1: Quality and risk-of-bias ratings

1. JBI Umbrella review consensus ratings

Items	Naidoo 2013 [15]	Goo et al., 2017 [41]	De Santos Moreno et al., 2023 [42]
1. Is the review question clearly and explicitly stated?	UNCLEAR	YES	YES
2. Were the inclusion criteria appropriate for the review question?	NO	YES	UNCLEAR
3. Was the search strategy appropriate?	YES	YES	UNCLEAR
4. Were the sources and resources used to search for studies adequate?	YES	YES	UNCLEAR
5. Were the criteria for appraising studies appropriate?	YES	YES	NO
6. Was critical appraisal conducted by two or more reviewers independently?	YES	UNCLEAR	YES
7. Were there methods to minimize errors in data extraction?	YES	UNCLEAR	YES
8. Were the methods used to combine studies appropriate?	YES	YES	YES
9. Was the likelihood of publication bias assessed?	NA	NA	NA
10. Were recommendations for policy and/or practice supported by the reported data?	YES	YES	UNCLEAR
11. Were the specific directives for new research appropriate?	YES	YES	UNCLEAR

2. ROBIS individual and consensus ratings

ROBIS Phase 2: IDENTIFYING CONCERNS WITH THE REVIEW PROCESS																										
Systematic Review	Rater	Domain 1. Study Eligibility Criteria						Domain 2. Identification & selection of studies						Domain 3. Data collection & Appraisal						Domain 4. Synthesis & Findings						
		1.1	1.2	1.3	1.4	1.5	Concerns	2.1	2.2	2.3	2.4	2.5	Concerns	3.1	3.2	3.3	3.4	3.5	Concerns	4.1	4.2	4.3	4.4	4.5	4.6	Concerns
Naidoo 2013 [15]	1	PY	PY	PN	PN	PN	HIGH	Y	NI	Y	PN	Y	LOW	Y	Y	Y	Y	Y	LOW	Y	Y	Y	Y	Y	N	LOW
	2	PY	PY	PN	PN	PN	HIGH	Y	NI	Y	PN	Y	LOW	Y	Y	Y	Y	Y	LOW	Y	Y	Y	Y	PY	N	LOW
	3	PY	PY	PN	PN	PN	HIGH	Y	NI	Y	PN	Y	LOW	Y	Y	Y	Y	Y	LOW	Y	Y	Y	Y	Y	N	LOW
Goo et al., 2017 [41]	1	PN	Y	Y	Y	Y	UNCLEAR	Y	Y	Y	PY	Y	LOW	NI	Y	Y	Y	PY	LOW	Y	NI	Y	Y	Y	N	LOW
	2	NI	Y	Y	Y	PN	UNCLEAR	Y	Y	Y	PY	Y	LOW	NI	Y	Y	Y	PY	LOW	Y	NI	Y	Y	Y	N	LOW
	3	PN	Y	Y	Y	N	UNCLEAR	Y	Y	Y	PY	Y	LOW	NI	Y	Y	Y	PY	LOW	Y	NI	Y	Y	Y	N	LOW
De Santos Moreno et al., 2023 [42]	1	PN	PN	N	PY	PY	HIGH	N	Y	PN	PY	Y	HIGH	Y	PN	PN	N	PY	HIGH	N	N	PY	PY	PN	N	HIGH
	2	PN	PN	PN	PY	PY	HIGH	N	PY	PN	PY	Y	HIGH	Y	PN	PY	N	PY	HIGH	N	N	Y	PY	PN	N	HIGH
	3	PN	PN	N	PY	PY	HIGH	N	Y	N	PN	Y	HIGH	Y	PN	PN	N	PY	HIGH	N	N	PY	PN	PN	PN	HIGH

ROBIS Phase 3: JUDGING RISK OF BIAS									
Systematic Review	Rater	Domain 1	Domain 2	Domain 3	Domain 4	RISK OF BIAS OF THE REVIEW			
						A	B	C	Overall risk
Naidoo 2013 [15]	1	HIGH	LOW	LOW	LOW	Y	Y	Y	LOW
	2	HIGH	LOW	LOW	LOW	Y	Y	Y	LOW
	3	HIGH	LOW	LOW	LOW	Y	Y	Y	LOW
Goo et al., 2017 [41]	1	UNCLEAR	LOW	LOW	LOW	Y	Y	Y	LOW
	2	UNCLEAR	LOW	LOW	LOW	Y	Y	Y	LOW
	3	UNCLEAR	LOW	LOW	LOW	Y	Y	Y	LOW
De Santos Moreno et al., 2023 [42]	1	HIGH	HIGH	HIGH	HIGH	PN	PN	Y	HIGH
	2	HIGH	HIGH	HIGH	HIGH	PN	PN	Y	HIGH
	3	HIGH	HIGH	HIGH	HIGH	PN	PN	Y	HIGH

Y: Yes; PY: Probably Yes; NI: No information; N: No; PN: Probably no

PHASE 3: Judging Risk of Bias of the Review, signalling questions:
A: Did the interpretation of findings address all of the concerns identified the in Phase 2 assessment?
B: Was the relevance of the identified studies to the review's research question appropriately considered?
C: Did the reviewers avoid emphasizing results on the basis of their statistical significance?
LOW risk of bias: The findings of the review are likely to be reliable. Phase 2 did not raise any concerns with the review process or concerns were appropriately considered in the review conclusions. The conclusions were supported by the evidence and included consideration of the relevance of the included studies.
HIGH risk of bias: One or more of the concerns raised during the Phase 2 assessment was not addressed in the review conclusions, the review conclusions were not supported by the evidence, or the conclusions did not consider the relevance of the included studies to the review question.
UNCLEAR risk of bias: There is insufficient information reported to make a judgement on risk of bias.

3. AGREE II ratings of clinical algorithm (Naidoo/Govender [24])

Domain	Item	Rater 1	Rater 2	Rater 3	Total	%	Section %
Scope	1. Objective	7	7	7	21	100	59/63 94%
	2. Question	6	6	5	17	81	
	3. Population	7	7	7	21	100	
Stakeholders	4. Professionals	7	7	7	21	100	52/63 82.5%
	5. Patients	3	4	3	10	48	
	6. Users defined	7	7	7	21	100	
Rigor	7. Evidence search	7	7	7	21	100	145/168 86%
	8. Criteria	7	7	7	21	100	
	9. Strengths and limitations	6	6	5	17	81	
	10. Recommendation formulation	7	7	6	20	95	
	11. Benefits and risks	6	6	6	18	86	
	12. Evidence linked	7	7	7	21	100	
	13. Expert review	7	7	7	21	100	
	14. Updating procedure	2	2	2	6	29	
Clarity	15. Recommendations	6	6	5	17	81	51/63 81%
	16. Management options	5	5	5	15	71	
	17. Key recommendations	7	6	6	19	90	
Applicability	18. Barriers and facilitators	5	6	4	15	71	50/84 59%
	19. Advice/tools	6	6	4	16	76	
	20. Resource implications	4	5	4	13	62	
	21. Monitoring/auditing	2	2	2	6	29	
Editorial independence	22. Funding independence	7	7	7	21	100	42/42 100%
	23. Competing interests	7	7	7	21	100	
Overall quality		6	6	6			18/21 86%
Recommendation		Yes, with modifications					

4: Mixed Methods Appraisal Tool (MMAT), version 2018

http://mixedmethodsappraisaltoolpublic.pbworks.com/w/file/fetch/127916259/MMAT_2018_criteria-manual_2018-08-01_ENG.pdf

		Methodological quality criteria					
**Category and study**		1	2	3	4	5	Comments by reviewers
**1. Qualitative**							
Govender & Joubert 2016[21]	Rater 1	Y	Y	Y	Can't tell	Y	No quotes used to substantiate comments - just a summary by the author
	Rater 2	Y	Y	Y	Can't tell	Y	
	Rater 3	Y	Y	Y	Can't tell	Y	
**3. Quantitative non-randomized**							
Segal et al., 2016 [23]	Rater 1	Y	Can't tell	Y	Y	Y	Limited psychometric properties of the muscle tone assessment
	Rater 2	Y	N	Y	Y	Y	
	Rater 3	Y	N	Y	Y	Y	
**4. Quantitative descriptive**							
Naidoo 2013b [18]	Rater 1	Y	Y	Y	Y	Y	Sample calculation adequate<br>Survey tool development excellent quality
	Rater 2	Y	Y	Y	Y	Y	
	Rater 3	Y	Y	Y	Y	Y	
Naidoo & Joubert 2013 [20]	Rater 1	Y	Y	Y	Y	Y	
	Rater 2	Y	Y	Y	Y	Y	
	Rater 3	Y	Y	Y	Y	Y	
**5. Mixed-methods**							
Govender & Joubert 2018 [24]	Rater 1	Y	Y	Y	Y	Y	
	Rater 2	Y	Y	Y	Y	Y	
	Rater 3	Y	Y	Y	Y	Y	

1. Qualitative 1.1. Is the qualitative approach appropriate to answer the research question? 1.2. Are the qualitative data collection methods adequate to address the research question? 1.3. Are the findings adequately derived from the data? 1.4. Is the interpretation of results sufficiently substantiated by data? 1.5. Is there coherence between qualitative data sources, collection, analysis and interpretation?
3. Quantitative nonrandomized 3.1. Are the participants representative of the target population? 3.2. Are measurements appropriate regarding both the outcome and intervention (or exposure)? 3.3. Are there complete outcome data? 3.4. Are the confounders accounted for in the design and analysis? 3.5. During the study period, is the intervention administered (or exposure occurred) as intended?
4. Quantitative descriptive 4.1. Is the sampling strategy relevant to address the research question? 4.2. Is the sample representative of the target population? 4.3. Are the measurements appropriate? 4.4. Is the risk of nonresponse bias low? 4.5. Is the statistical analysis appropriate to answer the research question?
5. Mixed methods 5.1. Is there an adequate rationale for using a mixed methods design to address the research question? 5.2. Are the different components of the study effectively integrated to answer the research question? 5.3. Are the outputs of the integration of qualitative and quantitative components adequately interpreted? 5.4. Are divergences and inconsistencies between quantitative and qualitative results adequately addressed? 5.5. Do the different components of the study adhere to the quality criteria of each tradition of the methods involved?

5. COSMIN reliability evidence ratings

Measure	Subjective	Hammersmith Infant Neurological Examination (HIINE)							
ROB Reliability	Wessel et al., 2013 [22]<br>n = 57<br>Inter-rater	Tedla et al., 2014 [72]<br>n = 31<br>Inter-rater<br>Global score	Tedla et al., 2014 [72]<br>n = 31<br>Inter-rater<br>Grades	Adıgüzel et al., 2022[77]<br>n = 35<br>Inter-rater<br>Global score	Romeo et al., 2022 [75]<br>n = 100<br>Inter-rater<br>Item scores	Tedla et al., 2014 [72]<br>n = 31<br>Intra-rater<br>Global score	Tedla et al., 2014 [72]<br>n = 31<br>Intra-rater<br>Grades	Harpster et al., 2022 [79]<br>n = 15<br>Intra-rater	Adıgüzel et al., 2022 [77]<br>n = 35<br>Intra-rater
1. Stability of patients	Very good	Very good	Very good	Very good	Doubtful	Very good	Very good	Doubtful	Very good
2. Time interval	Very good	Very good	Very good	Very good	Doubtful	Very good	Very good	Doubtful	Very good
3. Similarity of measurement condition	Very good	Adequate assignment	Adequate - assignment	Adequate Assignment	Doubtful	Adequate - from video	Adequate - from video	Adequate	Adequate - from video
4. Administration without knowledge	Adequate	Adequate Rater 1	Adequate Rater 1	Very good Rater 1	Doubtful	NA	NA	Very good	NA
5. Assignment without knowledge	Adequate	Adequate	Adequate	Very good	Doubtful	Adequate	Adequate	Very good	Very good
6. Other important flaws	Very good	Very good	Doubtful	Very good	Very good	Very good	Doubtful	Very good	Very good
Statistical									
7. Continuous scores: ICC or correlation?	NA	Adequate - no formula	ICC for dichotomous	Adequate - no formula	Adequate	Adequate - no formula	ICC for dichotomous	Adequate - no formula	Adequate - no formula
8. Ordinal scores: Weighted kappa?	NA	NA		NA	NA	NA	NA	NA	NA
9. Dichotomous/nominal unweighted kappa	Very good		No - doubtful	NA	NA	NA	No - doubtful	NA	NA
Final rating	Adequate	Adequate	Doubtful	Adequate	Doubtful	Adequate	Doubtful	Doubtful	Adequate

ICC: intraclass correlation coefficient; Grades: optimal/suboptimal scores; NA: question not appropriate for this study

6. COSMIN validity evidence ratings

Box 8 Criterion validity: Predictive and concurrent

Gold standards = CP diagnosis; Bayley; INFANIB; AIMS; Hypotonia (expert rater)

	Objective	Hammersmith Infant Neurological Examination (HINE)						
Statistical	Soucy et al., 2016 [14] Objective measures and expert rater n = 55	Tedla et al., 2014 [72] Global scores HINE and INFANIB n = 31	Tedla et al., 2014 [72] Optimal/suboptimal grade: HINE and Pediatrician n = 31	Romeo et al., 2016 [70] 7 studies HINE and CP diagnosis n = 2709	Romeo et al., 2022 [73] preterm infants HINE and MDI n = 1229	Romeo et al., 2021 [74] term infants HINE and MDI n = 446	Pietruszewski et al., 2022 [78] HINE and CP diagnosis n = 1389	Jansen Van Rensburg 2022 [71] High risk infants HINE and AIMS n = 100
1. Continuous scores: correlations or AUC calculated?	NA	ICC correlation not AUC	Used ICC for dichotomous		Very good	Very good	Very good	Very good
2. Dichotomous scores: sensitivity and specificity determined?	Very good	NA	No - Doubtful	Very good	Very good	Very good	Very good	Very good
Other								
3. Other important flaws in the design or statistical methods?	Very good	Question stats - Doubtful	Very good	Very good	Very good	Very good	Very good	Very good
COSMIN overall	Very good	Doubtful	Doubtful	Very good	Very good	Very good	Very good	Very good

AIMS: Alberta Infant Motor Scales; AUC: Area under the receiver operating curve; CP: cerebral palsy; HINE: Hammersmith Infant Neurological Examination; ICC: intraclass correlation coefficient; INFANIB: Infant Neurological International Battery; MDI: Mental Developmental Index (from Bayley II scales)

Box 9 Hypotheses testing for construct validity: Comparison of HINE with other instruments - convergent validity

Design Convergent	Uusitalo et al., 2021 [76] n = 174	Harpster et al., 2022 [79] n = 392	Harpster et al., 2022 [79] n = 392	Adıgüzel et al., 2022 [77] n = 35
1. Is it clear what the comparator measures?	WISC - IQ Very good	MRI - brain structure/damage Very good	GMA Very good	BSID III Cognitive, Language, Motor scales Very good
2. Measurement properties of the comparator sufficient?	Very good	Very good	Very good	Very good
Statistical				
3. Statistical method appropriate?	Very good	Very good	Very good	Very good
Other				
4. Any other important flaws in design or statistical methods?	Very good	Very good	Very good	Very good
Overall COSMIN	Very good	Very good	Very good	Very good

BSID III: Bayley Scales of Infant Development version III: GMA: Prechtl's General Movements Assessment; IQ: intelligence quotient; MRI: magnetic resonance imaging; WISC: Weschler Intelligence Scale for children 4th edition

References

14. Soucy, E.A.; Wessel, E.A.; Gau, F.; Albers, A.C.; Gutman, D.H.; Dunn, C.M. A pilot study for evaluation of hypotonia in children with neurofibromatosis type 1. *J Child Neurol* **2015**, *30,* 382-385 doi:10.1177/0883073814531823

15. Naidoo, P. Towards evidence-based practice - A systematic review of methods and tests used in the clinical assessment of hypotonia. *South African J Occup Ther.* **2013,** *43,* 2-8.

18. Naidoo, P. Current practices in the assessment of hypotonia in children. *South African J Occup Ther.* **2013,** *43,* 12-1.

20. Naidoo, P.; Joubert, R.W.E. Consensus on hypotonia via delphi process. *Indian J Pediatr.* **2013,** *80,* 641-650. doi:10.1007/s12098-013-1018-7

21. Govender, P.; Joubert, R.W.E. "Toning' up hypotonia assessment: A proposal and critique. *African J Disabil.* **2016,** *5,* 231: doi: 10.4102/ajod.v5i1.231

22. Wessel, L.E.; Albers, A.C.; Goutmann, D.H.; Dunn, C.M. The association between hypotonia and brain tumors in children with neurofibromatosis type 1. *J Child Neurol.* **2013,** *28,* 1664-1667. doi:10.1177/0883073812460918

23. Segal, I.; Peylan, T.; Sucre, J.; Levi, L.; Bassan, H. Relationship between central hypotonia and motor development in infants attending a high-risk neonatal neurology clinic. *Pediatr Phys Ther.* **2016,** *28,* 322-336. doi:10.1097/PEP.0000000000000265

24. Govender, P.; Joubert, R.W.E. Evidence-based clinical algorithm for hypotonia assessment: To pardon the errs. *Occup Ther Int.* **2018,** *89675,* 1-7. doi:10.1155/2018/8967572

70. Romeo, D.M.; Ricci, D.; Brogna, C.; Mercuri, E. Use of the Hammersmith Infant Neurological Examination in infanst with cerebral palsy: A critical review of the literature. *Dev Med Child Neurol.* **2016,** *58,* 240245. doi:10.1111/dmcn.12876

71. Jansen van Rensburg, E.A.; Burger, M; Unger, M. The predictive validity of Hammersmith Infant Neurological Examination versus Prechtl's General Movement Assessment with the Motor Optimality Score on gross motor outcomes in high-risk infants at 12-15 months corrected age: a descriptive study. MSc thesis 2022 https://scholar.sun.ac.za

72. Tedla, J.S.; Bajaj, A.; Joshua, A.M.; Kamath, G. Psychometric properties of Hammersmith Infant Neurological Examination in 12 months old high-risk infants: A cross-sectional study. *Indian J Physiother Occup Ther-An Int J.* **2014**, *8,* 169. doi:10.5958/j.0973-5674.8.2.081

73. Romeo, D.M.; Cowan, F.M.; Haataja, L.; Ricci, D.; Pede, E.; Gallini, F.; Cota, F.; Brogna, C.; Romeo, M.G.; Vento, G.; Mercuri, E. Hammersmith Infant Neurological Examination for infants born preterm: predicting outcomes other than cerebral palsy. *Dev Med Child Neurol.* **2021**, *63,* 939-946. doi:10.1111/dmcn.14768

74. Romeo, D.M.; Cowan, F.M.; Haataja, L.; Ricci, D.; Pede, E.; Gallini, F.; Cota, F.; Brogna, C.; Romeo, M.G.; Vento, G.; Mercuri, E. Hammersmith Infant Neurological Examination in infants born at term: Predicting outcome other than cerebral palsy. *Dev Med Child Neurol.* **2022**, *64,* 871-880. doi:10.1111/dmcn.15191
75. Romeo, D.M.; Apicella, M.; Velli, C.; Brogna, C.; Ricci, D.; Pede, E.; Sini, F.; Coratti, G.; Gallini, F.; Cota, F.; Bovis, F.; Vento, G.; Mercuri, E. Hammersmith Infant Neurological Examination in low-risk infants born very preterm: a longitudinal prospective study. *Dev Med Child Neurol.* **2022**, *64,* 863-870. doi:10.1111/dmcn.14873
76. Uusitalo, K.; Haataja, L.; Nyman, A.; Lehtonen, T, Setänen, S. Hammersmith Infant Neurological Examination and long-term cognitive outcome in children born very preterm. *Dev Med Child Neurol.* **2021,** *63,* 947-953.
77. Adıgüzel, H.; Ünal Sarıkabadayı, Y.; Apaydın, U.; Katırcı Kırmacı, Z.I.; Gücüyener, K.; Günel Karadeniz, P.; Elbasan, B.Turkish validity and reliability of the Hammersmith Infant Neurological Examination (HINE) with high-risk infant group: A preliminary study. *Turkish Arch Pediatr.* **2022,** *57,* 151-159. doi:10.5152/TurkArchPediatr.2022.21231
78. Pietruszewski, L.; Moore-Clingenpeel, M.; Moellering, G.C.J.; Lewandowski, D.; Batterson, N.; Maitre, N.L. Predictive value of the test of infant motor performance and the Hammersmith infant neurological examination for cerebral palsy in infants. *Early Hum Dev*. **2022**,*174*,105665. doi:10.1016/j.earlhumdev.2022.105665
79. Harpster, K.; Merhar, S.; Priyanka Illapani, V.S.; Peyton, C.; Kline-Fath, B.; Parikh, N.A. Associations Between Early Structural Magnetic Resonance Imaging, Hammersmith Infant Neurological Examination, and General Movements Assessment in Infants Born Very Preterm. *J Pediatr*. **2021**,*232*, 80-86.e2. doi:10.1016/j.jpeds.2020.12.056
